# Proof-of-Concept Machine Learning Framework for Arboviral Disease Classification Using Literature-Derived Synthetic Data: Methodological Development Preceding Clinical Validation

**DOI:** 10.3390/healthcare14020247

**Published:** 2026-01-19

**Authors:** Elí Cruz-Parada, Guillermina Vivar-Estudillo, Laura Pérez-Campos Mayoral, María Teresa Hernández-Huerta, Alma Dolores Pérez-Santiago, Carlos Romero-Diaz, Eduardo Pérez-Campos Mayoral, Iván A. García Montalvo, Lucia Martínez-Martínez, Héctor Martínez-Ruiz, Idarh Matadamas, Miriam Emily Avendaño-Villegas, Margarito Martínez Cruz, Hector Alejandro Cabrera-Fuentes, Aldo-Eleazar Pérez-Ramos, Eduardo Lorenzo Pérez-Campos, Carlos Mauricio Lastre-Domínguez

**Affiliations:** 1 División de Estudios de Posgrado e Investigación, Instituto Tecnológico de Oaxaca, Tecnológico Nacional de México, Oaxaca de Juárez C.P. 68030, Mexico; eli.cruz.parada@gmail.com (E.C.-P.); aperez_santiago@hotmail.com (A.D.P.-S.); carlos.rom.74he@gmail.com (C.R.-D.); ivan.garcia@itoaxaca.edu.mx (I.A.G.M.); idarhmatadamas@gmail.com (I.M.); e_mily_3@hotmail.com (M.E.A.-V.); martinezcu9@hotmail.com (M.M.C.); dr.aldo@itoaxaca.edu.mx (A.-E.P.-R.); 2 Facultad de Sistemas Biológicos e Innovación Tecnológica, Universidad Autónoma Benito Juárez de Oaxaca, Oaxaca de Juárez C.P. 68120, Mexico; gvivar.cat@uabjo.mx; 3 Centro de Investigación Facultad de Medicina UNAM-UABJO, Facultad de Medicina y Cirugía, Universidad Autónoma “Benito Juárez” de Oaxaca, Oaxaca de Juárez C.P. 68020, Mexico; lperezcampos.fmc@uabjo.mx (L.P.-C.M.); eperezcampos.fmc@uabjo.mx (E.P.-C.M.); lumartin1969@yahoo.com.mx (L.M.-M.); hmartinez.fmc@uabjo.mx (H.M.-R.); hafuentes@iau.edu.sa (H.A.C.-F.); 4 Secretaria de Ciencia, Humanidades, Tecnología e Innovación (SECIHTI), Facultad de Medicina y Cirugía, Universidad Autónoma “Benito Juárez” de Oaxaca, Oaxaca de Juárez C.P. 68020, Mexico; mthernandez@secihti.mx; 5 R&D Group, Vice Presidency Scientific Research & Innovation, Imam Abdulrahman Bin Faisal University (IAU), Dammam P.O. Box 1982, Saudi Arabia; 6 División de Estudios de Posgrado e Investigación, Instituto Tecnológico de Tijuana, Tecnológico Nacional de México, Tijuana C.P. 22414, Mexico

**Keywords:** arboviral disease, machine learning, classification, statistical analysis, Dengue, Zika, Chikungunya

## Abstract

**Highlights:**

**What are the main findings?**

- Extraction and selection of features from 67 symptoms using binary coding.
- Model of classification for arboviral diseases using different methods based on machine learning and deep learning.

**What are the implications of the main findings?**

- Conducts rigorous statistical analysis of data to identify symptoms more prevalent for different arboviral diseases using Odds Ratio and Chi-square.
- Performance evaluation using metrics such as F1-score, accuracy, precision, sensitivity, specificity, AUC-ROC, and Cohen’s kappa.

**Abstract:**

**Background/Objectives**: Arboviral diseases share common vectors, geographic distribution, and symptoms. Developing Machine Learning diagnostic tools for co-circulating arboviral diseases faces data-scarcity challenges. This study aimed to demonstrate that proof of concept using synthetic data can establish computational feasibility and guide future real-world validation efforts. **Methods**: We assembled a synthetic dataset of 28,000 records, with 7000 for each disease—Dengue, Zika, and Chikungunya—plus Influenza as a negative control. These records were obtained from the existing literature. A binary matrix with 67 symptoms was created for detailed statistical analysis using Odds Ratios, Chi-Square, and symptom-specific conditional prevalence to validate the clinical relevance of the simulated data. This dataset was used to train and evaluate various algorithms, including Multi-Layer Perceptron (MLP), Narrow Neural Network (NN), Quadratic Support Vector Machine (QSVM), and Bagged Tree (BT), employing multiple performance metrics: accuracy, precision, sensitivity, specificity, F1-score, AUC-ROC, and Cohen’s kappa coefficient. **Results**: The dataset aligns with the PAHO guidelines. Similar findings are observed in other arboviral databases, confirming the validity of the synthetic dataset. A notable performance across all evaluated metrics was observed. The NN model achieved an overall accuracy of 0.92 and an AUC above 0.98, with precision, sensitivity, and specificity values exceeding 0.85, and an average Uniform Cohen’s Kappa of 0.89, highlighting its ability to reliably distinguish between Dengue and Influenza, with a slight decrease between Zika and Chikungunya. **Conclusions**: These models could accelerate early diagnosis of arboviral diseases by leveraging encoded symptom features for Machine Learning and Deep Learning approaches, serving as a support tool in regions with limited healthcare access without replacing clinical medical expertise.

## 1. Introduction

The challenge of early diagnosis for diseases such as Dengue, Zika, and Chikungunya is a crucial issue in healthcare; differentiating between them is vital for proper treatment and prognosis, especially in outbreak areas. Symptomatic ambiguity during the febrile phase highlights the need to develop a decision-support tool to enable quick and accurate decisions. Therefore, this work aims to advance this field by designing and evaluating an in silico study based on Machine Learning (ML) and Deep Learning (DL) for the early and robust differentiation of these three arboviruses (arthropod-borne viruses), including Influenza as a negative control.

In general, arboviral diseases are transmitted to humans by other living organisms, typically insects such as mosquitoes, fleas, lice, and ticks. When a vector becomes infected, it transmits the pathogen through a bite or sting. These pathogens can be viruses, bacteria, or parasites; vectors usually remain infectious throughout their lives. The geographic distribution of these vectors and the diseases they transmit depends on various factors, including demographic, environmental, and social elements. For example, climate change is predicted to expand the habitat of Aedes mosquitoes [1,2]. These mosquitoes are vectors of arboviruses and are responsible for diseases such as Dengue, Zika, and Chikungunya, among others.

The arboviral diseases analyzed in this study—Dengue, Zika, and Chikungunya—share symptomatic presentations, sociodemographic distribution, and even outbreak seasons, making early diagnosis invaluable. To illustrate their similarities and differences, a general definition of the pathologies under study will be provided. In addition, information on Influenza is also, used here as a negative control.

### 1.1. Dengue Fever

Dengue is a febrile illness caused by a virus of the Flaviviridae genus, called Dengue virus (DENV), which primarily affects tropical and subtropical regions worldwide [3]. This virus has four serotypes: DENV-1, DENV-2, DENV-3, and DENV-4, all of which affect humans [4]. This disease is generally transmitted by the bite of female mosquitoes of the *Aedes* genus, specifically the aegypti species, followed by the albopictus species [5]. Dengue is primarily asymptomatic; if symptoms appear, they typically improve within one to two weeks. However, in some patients, these symptoms worsen and require hospitalization, as they can lead to death, although the case fatality rate is very low [6]. The World Health Organization (WHO) has reported a significant increase in the incidence rate worldwide, increasing from 505,430 in 2000 to 14.6 million in 2024. The Pan American Health Organization (PAHO), in its epidemiological report for the Americas region through week 33 (W1-33) of 2025, reports 3,704,813 suspected cases, of which 40% were laboratory-confirmed, and 1865 deaths, with a case fatality rate of 0.05% [7]. The most common symptoms include a high fever (above 40 °C/104 °F), headache, muscle and joint pain, nausea, vomiting, and a skin rash. The distinctive symptoms and signs of Dengue may be slightly different in the under-18 age population, e.g., the prevalence difference. Cases of Dengue have been observed where the prevalence of symptoms was 19% abdominal pain, 41% leukopenia, 16% nausea, 22% vomiting, and 42% basophilia [1].

In 2009, the WHO and the Program for Research and Training in Tropical Diseases, a collaborative effort with reviewers and contributors from both within and outside the organization, published “Dengue Guidelines for Diagnosis, Treatment, and Control” [8]. Symptomatic infections of the disease were classified into two categories: severe and non-severe dengue. Non-severe dengue is further divided into Dengue without warning signs and Dengue with warning signs for practical use by treating physicians. The progression of the disease consists of three phases: the febrile phase, the critical phase (where warning signs may appear), and the recovery phase. Diagnosis is usually clinical, with laboratory tests used only for confirmation [9]; however, limited diagnostic expertise and lack of access to public health services can contribute to delayed patient care [10]. Currently, there is no specific treatment; thus, pain relief is usually achieved with medications such as paracetamol. Nonsteroidal anti-inflammatory drugs (NSAIDs) are not recommended because they increase the risk of bleeding [11].

### 1.2. Zika Fever

Zika is a disease caused by the Zika virus (ZIKV) of the Flaviviridae genus. It is transmitted through the bite of mosquitoes of the genus *Aedes*, primarily Aedes aegypti, although it has been isolated in different species of the same genus [12]. It affects tropical and subtropical areas of the planet, as does DENV. In 2022, a study compiling evidence of autochthonous transmission in several countries was published [13]. Various studies confirm sexual transmission and perinatal vertical transmission (mother-to-child) of the disease [14]. Like Dengue, most infected individuals are asymptomatic [15]; however, if symptoms do occur, they generally last 2 to 7 days. Nevertheless, Zika has been linked to Guillain-Barré syndrome [16], as well as microcephaly and malformations in pregnancy [17,18]. In 2024, the PAHO recorded 44,490 cases, but only 2117 were laboratory-confirmed; no deaths were reported [19]. According to the PAHO publication “Case definitions, clinical classification, and stages of Dengue, Chikungunya, and Zika” [4], symptoms associated with Zika include sudden-onset rash, itching, conjunctivitis (non-purulent), muscle and joint pain, periarticular edema, and fever; of these, the most significant symptoms have been rash and conjunctivitis [2]. A confirmed case is defined only as one with a confirmatory laboratory test. Its diagnosis, like that of Dengue, is primarily clinical, which makes it challenging due to the high rate of asymptomatic individuals. Lack of detection does not imply that it is not circulating. It has been suggested that the methodology to determine the asymptomatic prevalence of this disease should be improved [20]. Research into specific treatments is ongoing; therefore, only rest and the use of analgesics and antipyretics are recommended to treat the pathology, avoiding the use of NSAIDs until Dengue is ruled out.

### 1.3. Chikungunya Fever

Chikungunya is a febrile illness caused by the Chikungunya virus (CHIKV), a member of the Alphavirus genus within the Togaviridae family. Cases have been reported in areas where Dengue and Zika are also prevalent [21]. This disease is transmitted by the bite of *Aedes* aegypti and albopictus mosquitoes [22]. From 2014 to 2024, the PAHO recorded 1,638,892 cases in the Americas, of which 888,475 (54%) were laboratory-confirmed [23]. Its symptoms include a sudden high fever (39 °C/102 °F) associated with severe joint and/or muscle pain, which is debilitating and disabling. These symptoms usually last a few days; however, joint pain can last for weeks, months, or even years [24]. The most significant symptoms have been reported to be arthralgia, photophobia, and dizziness. In addition, there is no leukopenia and no papular rash. Furthermore, the symptoms already mentioned include joint swelling, rash, headache, and nausea. Some patients with suspected Chikungunya may experience neurological, cardiovascular, dermatological, ophthalmological, hepatic, renal, respiratory, hematological, and other complications; therefore, the PAHO publication [25] classifies it into three phases: chikungunya, chikungunya with extra-articular manifestations, and severe chikungunya. In turn, depending on the duration of symptoms, it is defined in three phases: acute (symptoms last up to two weeks), post-acute (symptoms last until the end of the third month, either continuously or intermittently), and chronic (joint symptoms extend for more than three months). Although serious complications are rare, it has been observed that it can be a fatal outcome in children under one year of age and older adults with comorbidities [26]. The diagnosis is clinical; therefore, an infection may go unnoticed or be misdiagnosed if mild symptoms are present [27]. This disease has no specific treatment, although two vaccines are already available [28]; however, one of them was suspended in August 2025 by the U.S. Food and Drug Administration (FDA). Its treatment usually focuses on pain and fever relief; thus, analgesics and antipyretics are recommended, but as with Zika, it is suggested to avoid the use of NSAIDs until Dengue infection is ruled out. The three diseases studied here share a common vector, geographic distribution, some symptoms, and a febrile onset; consequently, their diagnosis remains complex [29].

### 1.4. Influenza

Influenza is the negative control in this investigation. Given that the pilot tool can only differentiate among the three arboviral diseases, it was decided to add Influenza because a patient may not present any of the arboviral diseases. Influenza is a respiratory disease caused by a virus from the Orthomyxoviridae family, of which there are four types: A, B, C, and D. Types A and B are most common and cause seasonal epidemics, with type A being the most relevant to humans due to its high mutation capacity [30]. Some of these viruses are specific to different species [31]. Influenza usually has a higher incidence during the colder months of the year, although it can also occur out of season. According to data from the WHO, about one billion cases are reported annually, of which between three and five million are severe [32]. In humans, it is primarily transmitted through droplets when sneezing, coughing, or talking. Symptoms include fever or feverish feeling, cough, sore throat, runny nose, muscle aches, headaches, and fatigue. It should be noted that not all infected people present symptoms [33]. Its clinical picture can be mild to severe, and it can sometimes cause death [34]. Most people who contract the disease recover within a few days or up to two weeks; however, complications such as pneumonia can develop and be life-threatening [35], especially in immunocompromised individuals [36]. Its clinical diagnosis makes Influenza caused by a type A virus practically indistinguishable from that caused by a type B virus. Since Influenza is a disease like other respiratory tract viruses, laboratory tests are necessary for confirmation. Its treatment does not require special measures, only symptomatic management, such as fever control, hydration, and rest. Vaccination is the best means of prevention to avoid severe cases. Currently, Influenza vaccination is offered in 41 countries and territories in the Americas, according to PAHO [37].

Despite the biological differences among the viruses mentioned, their initial clinical presentation converges on a nonspecific febrile syndrome that challenges the diagnostic ability of healthcare personnel, especially in resource-limited areas. This symptomatic ambiguity not only delays proper clinical management but also complicates epidemiological surveillance in regions where these diseases coexist.

This work presents a method for classifying arboviral diseases using Machine Learning (ML) algorithms and a rigorous statistical analysis of coded symptoms based on a synthetic dataset of confirmed cases from previous studies. Unlike previous approaches, Influenza is included as a negative control, enabling the model to identify when a patient does not have an arboviral disease. While characteristic symptoms typically emerge as the disease progresses, the focus is on early detection to improve follow-up and prognosis. This tool could support healthcare professionals during initial diagnosis, especially when laboratory confirmation is unavailable. The main findings of this study are as follows:

- The implementation of a binary matrix representing 67 symptoms associated with the examined diseases is designed to generalize the diagnostic process and mitigate issues arising from imbalanced datasets.
- The application of a robust statistical analysis to clearly understand the data’s dimensional structure and symptom prevalence using the Odds Ratio and Chi-square.
- The performance of ML and DL models is compared, accompanied by a comprehensive evaluation to assess their predictive capabilities utilizing metrics such as accuracy, precision, sensitivity, specificity, F1-score, AUC-ROC, and Cohen’s kappa coefficient.

## 2. Related Work

The search for technological solutions for predicting tropical diseases has gained traction over the past decade, driven by the digitization of clinical records and increased computing power. To place our proposal within the current context, it is essential to review how the scientific community has addressed the unbalanced and complex nature of arboviral clinical data.

In recent decades, the scientific community has developed technological solutions for automatically predicting tropical diseases, with several studies reported in the literature. For example, in a classification study [38], the authors demonstrated the effectiveness of Random Forest and Decision Tree techniques for the differential classification of Dengue, Zika, and Chikungunya, highlighting the potential of ML to discriminate arboviral diseases. The affirmation rate especially increases when based on the criteria established in the PAHO differential diagnosis guideline [25]. However, given the complexity of data collection and the tendency toward unbalanced datasets, the research team used Bootstrap Resampling Techniques to balance and expand their dataset from 150 to 267 records.

Another study, based on various ML and DL algorithms, most notably the Long Short-Term Memory (LSTM) network [39], addressed the challenge of small datasets in arboviral infections (534 and 98 records) by employing transfer learning to improve the models’ predictive performance. The team underlines that the use of these techniques is intended to improve class distribution. Another research team leverages transfer learning combined with the Analytic Hierarchy Process (AHP) to improve predictions of infectious disease outbreaks [40], demonstrating advanced methodological integration. A vast dataset of Dengue outbreaks was used to train predictive models for Zika and Chikungunya outbreaks. Similarly, to counteract the imbalance in the datasets, the Synthetic Minority Over-Sampling technique was used. Another point of view on the problem is the observation of vector behavior. A research team applied an ML model to predict the habitat suitability of Aedes albopictus [41], providing surveillance tools for the prevention of vector-borne diseases. Similarly, another interesting study implements models for the spatiotemporal analysis of arboviral diseases [42]; they mention that, although municipal-level datasets were used, individual or household-level factors that may influence vector distribution should be considered.

Likewise, there are different ways to address the same problem, e.g., innovative approaches using deployments of Wolbachia-infected mosquitoes have demonstrated effectiveness in reducing the incidence of Dengue and other arboviruses [43]. To observe this effectiveness, a research team implemented a Matrix-Assisted Laser Desorption Ionization Time-of-Flight (MALDI-TOF) coupled with ML techniques [44], concluding that the inclusion of ML in molecular detection techniques improves their performance. This is an attempt to control the vector. More recently, a comprehensive 18-year prospective cohort study compared the clinical characteristics of Dengue, Zika, and Chikungunya among children in Nicaragua [45], providing valuable longitudinal data on the pediatric prognosis of these diseases.

On another note, some of these diseases have a high rate of asymptomatic or afebrile infection; therefore, it would be extremely useful to use this data as part of a more complex model. Whatever the approach, the prevention, control, and monitoring of vector-borne diseases must be targeted, not to mention that this could be improved. For example, different age groups could be considered, as diseases can present different behaviors [45].

Therefore, all these advances require rigorous assessment metrics and statistical frameworks, as emphasized by Rainio et al. [46], particularly given the critical importance of interpretability and accuracy in biomedical time-series analysis [47], as well as the need to move beyond traditional assessment practices [48] toward more probabilistic reasoning approaches [49,50] to achieve diagnostic excellence. Although the methods for confirming these diseases, that is, laboratory tests, have proven to be highly reliable, their accessibility can be limited in hard-to-reach areas, emphasizing that the diagnosis is usually purely clinical, which relies on the experience and expertise of health professionals. Initially, in the febrile stage of Dengue, Zika, and Chikungunya, these diseases may go unnoticed; a fever at first glance can be interpreted as a flu-like syndrome [51,52]. Therefore, a computational tool based on the total symptom count could prove promising in distinguishing between the three diseases [53].

We have identified that the scarcity of multivariable data and class imbalance restricts the scope of existing diagnostic tools. In response, our methodology concentrates on a matrix of 67 specific symptoms derived from reported clinical evidence. This approach enables Machine Learning and Deep Learning models to function on a robust statistical foundation, utilizing metrics such as Odds Ratio and Chi-square to ensure that symptom prevalence informs the system’s predictive capabilities.

## 3. Methodology

**Figure 1:**
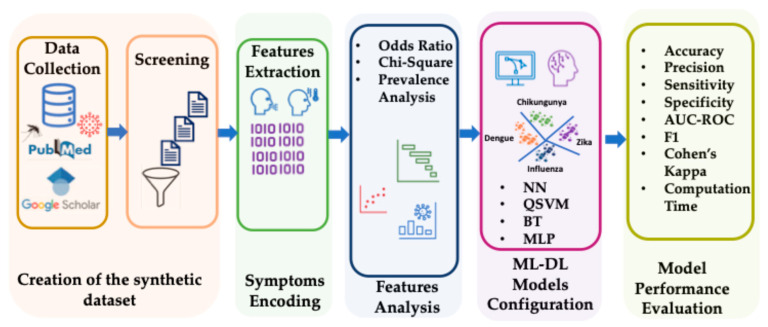
Flowchart of the research process. A graphical representation of the pipeline that was used in this study. PubMed, Public Medline; ML-DL, Machine Learning-Deep Learning; NN, Narrow Neural Network; QSVM, Quadratic Support Vector Machine; BT, Bagged Tree. MLP, Multi-Layer Perceptron; AUC-ROC, Area Under the Curve-Receiver Operating Characteristic curve; F1, harmonic mean of precision and sensitivity.

This section describes the research process, which is summarized in the following points: 1. Data collection, searching the dataset in recognized peer-reviewed publications. 2. Data extraction and screening, collecting data that will form the dataset for model training. 3. Data coding, transforming this dataset into a binary matrix that facilitates model learning. 4. Feature analysis, statistically exploring the variables in search of anomalies. 5. AI models, training, testing, and selecting different ML and DL models appropriate for the task. 6. Performance evaluation, once the models have been selected and several iterations are executed, their performance and reliability will be observed. This process is graphically represented in Figure 1. Each process is described below.

### 3.1. Creation of the Synthetic Dataset

A comprehensive search was conducted in the PubMed and Google Scholar datasets between February 2024 and May 2025. The following keywords and MeSH (Medical Subject Headings) terms were used in the PubMed and Google Scholar search engines: For Dengue, the combination “DENGUE”, “PREVALENCE”, “CLINICAL SYMPTOMS” was used. For Zika, Chikungunya, and Influenza, the same combination was used, except for the disease name, e.g., for Zika, “ZIKA”, “PREVALENCE”, “CLINICAL SYMPTOMS”. The following search criteria were applied to select articles for the four diseases included in this study: (1) The study must report at least 20 patients diagnosed and confirmed with the disease in question. (2) The study must report all symptoms observed in the patients. (3) Symptoms must have been observed within the first 5 days of the illness. (4) At least four symptoms must have been observed. (5) Patients selected for the dataset did not present with coinfections or reinfections.

Sensitive data, such as age, sex, nationality, socioeconomic status, income, or the medical unit where patients were treated, were not collected. Therefore, only the number of patients observed and their individual symptoms were taken from the selected studies. When a study presented laboratory data associated with the patients and the disease in question, these were considered symptoms of the disease, e.g., studies showing a white blood cell count < 4000 cells/mm^3^ were marked as Leukopenia present.

The data were extracted from various articles published in journals from several countries. Retrospective data from diverse geographic areas can provide a general understanding of the disease’s behavior and, therefore, broaden its potential applications. The dataset is available in a publicly accessible data repository [54]. Appendix A includes a table (Table A1) detailing the data extracted from scientific articles. It cites the studies used to create the synthetic dataset, highlights the most common symptom types, specifies the data sources in the articles, and indicates their locations. The symptoms presented by each observed patient were identified and transformed into a binary matrix, where “1” indicates presence and “0” indicates absence. The purpose of this transformation is to standardize the types of data collected across studies; therefore, all continuous data, such as body temperature readings, and nominal data, such as symptom or sign severity, were converted to binary data. During this data collection and transformation, preprocessing was performed, including the search for outliers, labeling errors, and duplicate records—errors that are likely to occur with manual data collection. The objective is to ensure the quality of the newly created synthetic dataset.

### 3.2. Symptoms Encoding

Following data collection, a total of 22,379 patients with Dengue were recorded. It is important to note that Dengue patients did not include those classified as severe Dengue, but did include those with Dengue without warning signs and Dengue with warning signs. Additionally, there were 7135 patients with Zika, 7959 patients with Chikungunya, and 10,741 patients with Influenza, including cases of Influenza A, Influenza B, or seasonal Influenza. Among the four diseases, up to 67 different symptoms were observed, and 12 symptoms were shared across all four. The collected data were converted to binary values, creating a binary dataset for each patient. Equation (1) represents the mapping of the dataset. For m patients and n symptoms, the matrix X is defined as follows:

(1) $$X=\left(\begin{array}{c} \begin{array}{cc} x_{1,1} & x_{1,2}\\ x_{2,1} & x_{2,2} \end{array}\\ \begin{array}{cc} \vdots & \vdots \\ x_{m,1}\; & x_{m,2} \end{array}\; \end{array}\;\;\begin{array}{c} \begin{array}{cc} \ldots & x_{1,n}\\ \ldots & x_{2,n} \end{array}\\ \begin{array}{cc} \ddots & \vdots \\ \ldots \; & x_{m,n} \end{array}\; \end{array}\right)$$

where $x_{ij}$ is the binary encoding for patient i and symptom j, as seen in Equation (2).

(2) $$x_{ij}=\left\{\begin{array}{c} 1\\ 0 \end{array}\right.\begin{array}{c} \;\mathrm{if}\;\;\mathrm{symptom}\;j\;\mathrm{is}\;\mathrm{present}\;\mathrm{in}\;\mathrm{patient}\;i\\ \mathrm{if}\;\mathrm{symptom}\;j\;\mathrm{is}\;\mathrm{absent}\;\mathrm{in}\;\mathrm{patient}\;i \end{array}$$

To ensure all patients had the same number of symptoms, symptoms from other diseases were included, even if they were not specific to the patient or the disease. Therefore, each patient, now called a record, has 67 symptoms, now referred to as features. The coded dataset with a total of 48,214 records is in an open-access repository [54], which is linked to a “Data Descriptor” article currently under review. The 67 observed symptoms can be seen in Table 1, classified by the affected physiological system.

However, given the imbalance in the number of records among the diseases (now classes), the number of records from the least common class (Zika, 7135 records) was used as a reference to randomly select 7000 records per class. Consequently, a dataset with 28,000 records, four classes, and 67 features per record was obtained. This balanced dataset was subjected to various statistical analyses to validate its behavior, that is, to determine if it is analogous to a real-world dataset. Similarly, the statistical analyses allowed us to establish which features showed a statistically significant association with each class, as well as to observe the predictive impact on the ML models that could represent the features. The statistical analyses applied were the following: an Odds Ratio analysis, a Feature Importance analysis using the Chi-square algorithm, and a Symptom Prevalence analysis.

### 3.3. Statistical Analysis Techniques

#### 3.3.1. Odds Ratio Analysis

The statistical analyses were conducted with the purpose of thoroughly understanding the data contained in the synthetic dataset created for this study. This approach ensures the identification of potential outliers, labeling errors, missing values, or duplicated instances—factors that commonly hinder model performance. Additionally, these analyses allow for the assessment of data quality, since a high-quality dataset significantly enhances the models’ ability to achieve their intended objectives.

To determine whether the synthetic dataset generated from the studies presented anomalies and to assess the strength of association between symptoms and pathologies, the Odds Ratio (OR) was calculated [55]. This allows us to identify which symptoms are most strongly associated with each pathology. A 95% confidence interval (CI) and Fisher’s exact test were used to assess whether the association is statistically significant. For two variables that can be symptoms or pathologies, $j_{1}$ and $j_{2}$, derived from m × n data matrix X, requires the frequency count described in Table 2. The calculation of $OR_{j_{1},j_{2}}$ requires summarizing the m patient observations into a 2 × 2 contingency table defined by four fundamental frequency counts, a, b, c, and d.

$I_{\left\{A\right\}}$ is the indicator function, and m is the total number of patients. Therefore, the OR Equation (3) is based on the previous contingency table:

(3) $$OR=\frac{\left(a\times d\right)}{\left(b\times c\right)}$$

OR displays positive values from 0 to infinity. To interpret the OR function, focus on the number 1. If the OR equals 1, there is no association; if OR > 1, it suggests a positive association, i.e., a symptom that is more frequent in patients with the target pathology; and if OR < 1, it indicates a negative association, a less frequent symptom, which could even be an indicator of another disease. However, to better visualize the magnitude of the OR, a natural logarithm (log) was applied, as it centers the values on the number 0, making it easier to distinguish its direction and magnitude. The logarithmic transformation generated a range of values between negative infinity and positive infinity, allowing for a better understanding of the results.

#### 3.3.2. Chi-Square Analysis

From an ML perspective, a common practice is to discard features with very low or insignificant values when preparing a predictive model, as they appear to be uninformative. These values can be calculated using different statistical measures. Here, a Feature Importance test was performed using the Chi-square test. The Feature Importance test is a measure that seeks to gauge the influence on the prediction of an ML model. Its calculation was based on Equation (4):

(4) $$\chi^{2}=\sum_{i=1}^{m}\sum_{j=1}^{n}\frac{{\left(O_{ij}-E_{ij}\right)}^{2}}{E_{ij}}$$

where $\chi^{2}$ is the chi-square value for the i-th patient and j-th symptom, $O_{ij}$ is the observed frequency in the matrix X as given by Equation (1), $\varepsilon_{ij}$ is the expected frequency, m denotes the number of patients, and n denotes the number of symptoms.

A higher score indicates a likely greater impact on the final prediction, while a very low score, or one close to zero, may lead to the removal of the feature to simplify the model due to its low predictive impact. However, a higher score does not necessarily mean that it is reflected in the final model prediction.

#### 3.3.3. Symptom Prevalence Analysis

Another test used was a Symptom Prevalence analysis. For this, the prevalence of symptoms by disease and overall was first calculated in Equation (5).

(5) $$Prevalence_{j|k}=\frac{\sum_{i=1}^{m}x_{i,j}y_{i,k}}{\sum_{i=1}^{m}y_{i,k}}$$

where $Prevalence_{j|k}$ is the conditional prevalence of the symptom j given pathology k, $x_{i,j}$ is the binary encoding defined by Equation (2) and $y_{i,k}$ is the ith patient with pathology k, with k={1,2,3,4} representing the four associated pathologies.

The use of the different types of statistical analyses described above allowed us to create a logic for selecting features. Odds Ratio identified the features with the highest degree of association to each class (*p* < 0.05), the Features Importance test provided the predictive capacity of the features, and the Symptom Prevalence analysis allowed us to observe the particular features of each class. Consequently, it was possible to create a subset with a reduced number of features; this subset contains twenty features. However, when working with medical data, feature reduction must also be approved by clinical validation from medical experts, beyond statistical studies. Therefore, we used this subset of data with 20 features to include an additional analysis to see if feature reduction affects ML models and especially DL models, since the complex architectures of DL models and Neural Networks seek to capture relationships in features, even if there is minimal presence of certain features, relationships that simple statistical tests might ignore.

### 3.4. Machine Learning and Deep Learning Models Configuration

During the research process, several ML algorithms and one DL algorithm were trained and tested to observe and evaluate their different behaviors. Algorithms tested but not shown in this study included Linear Discriminant Analysis, Medium K-Nearest Neighbors, and Random Under-Sampling Boosting Trees. The algorithms defined here are those that showed the best performance and will be briefly explained.

The trained and tested algorithms were the following: a Multi-Layer Perceptron (MLP), a Narrow Neural Network (NN), a Quadratic Support Vector Machine (QSVM), and a Bagged Tree (BT). MLP has a DL architecture, while NN, QSVM, and BT are classic ML algorithms. All models were trained and tested using supervised learning. This means that the training and validation test datasets contained the sample data and labels. In this way, the algorithms, following their own design principles, sought to identify the relationships between the features and the labels.

Regarding the models’ hyperparameters—the external configurations that the models contain and control the learning process before training—these were adjusted using Bayesian Optimization. This technique seeks the best configuration by using a probabilistic model that predicts performance and balances the hyperparameters in an objective function, which in this case was calculated based on the loss function.

#### 3.4.1. Multi-Layer Perceptron

A Multi-Layer Perceptron (MLP) is a DL artificial neural network widely used for classification, regression, and pattern recognition tasks. It consists of multiple interconnected layers that process data through their different nodes or neurons. Each node transforms the values through weight optimizations and nonlinear activation functions. In the end, the output layer receives the values from the last hidden layer and transforms them again to show the output values. These layers can be based on biological architectures [56]. MLP is trained by Backpropagation, minimizes the Cross-Entropy Loss function, and supports multiclass classification by applying Softmax as the output function [57]. Applications include image classification, medical diagnosis, and vision tasks [58].

The hyperparameters were as follows: the MLP was built with an input layer of 67 nodes (FeatureInputLayer), five fully connected hidden layers, ReLU activation function, four output layers (classes), Softmax function for multiclass, and was trained at different numbers of epochs ranging from 100 to 1000, with an initial learning rate of 0.01. An “ADAM” optimizer and a “crossentropy” loss function were used.

#### 3.4.2. Narrow Neural Network

A Narrow Neural Network (NN) is a type of artificial neural network with a small number of neurons or nodes per layer, typically even fewer than the number of dimensions of the input. Like MLP, it is a layered system that connects the data of its nodes using weights that adjust as the algorithm learns from the data. However, the difference between the MLP and the NN lies in the fact that the NN uses continuous sigmoid nonlinearities in the hidden units, while the MLP employs step function nonlinearities, which implies a fundamental role in the training of the networks [59]. Its applications include classification, regression, speech recognition, and image recognition. Its architecture is based on the brain [60].

Unlike MLP, the NN was not built with a DL architecture, although it shares certain parameters. Its specifications include a ReLU activation function, an iteration limit of 1000, a regularization rate (Lambda) of 0, and a fully connected hidden layer of 10 nodes.

#### 3.4.3. Support Vector Machine

Support Vector Machines (SVMs) are rigorous ML algorithms used for regression and classification tasks. Their mathematical principle is to establish an optimal hyperplane that separates all data points of a class from those of the other with the maximum margin. Margin refers to the width of the slab parallel to the hyperplane that does not contain internal data points. Support vectors are the data points closest to the separating hyperplane; these points lie on the edge of the slab [61]. SVMs are useful for high-dimensional data; for example, when the data is nonlinear, they use kernel functions to project these high-dimensional spaces, making their separation possible [62]. This flexibility allows for a wide applicability of the ML model; its application has been observed in healthcare [63].

The configuration of QSVM was a polynomial kernel function of second order, automatic kernel scaling, a box-constrained optimization of 1, and a multiclass encoding of “One vs. One.”

#### 3.4.4. Bagged Tree

A Bagged Decision Tree (BT) is an ML technique that improves the stability and accuracy of decision trees. This technique, also known as bootstrap aggregating, aims to prevent overfitting and reduce variance, thus providing greater model reliability than a single decision tree. To apply bagging to a decision tree, multiple bootstrap replicas of the dataset are generated, different random samples of the data are trained on multiple decision trees, and the predictions are combined [64]. Furthermore, each tree can randomly select predictors for each decision split, a technique called Random Forest [65], which improves the accuracy of the trees in bagging. It is used in classification and regression tasks, and its application has been observed in medical diagnosis [66].

Regarding BT, a bagging meta-estimator was added to this algorithm to improve the model. Its parameters were a maximum number of splits: 22,399, “bag” meta-estimator, learning cycles at 30, and a number of learners: 30.

### 3.5. Classification Metrics and Evaluation Rationale

Regarding the evaluation of models, many different performance metrics are often presented, as several are often recommended for a particular goal or case [46], e.g., for unbalanced classes. Ultimately, they are all related to each other in one way or another. Some studies argue that high accuracy of ML models denotes low interpretability in the medical field; black-box models are mentioned [47]. Therefore, no metric can accurately summarize a model’s performance [48]; here, we showed the general behavior of trained models, through which we can observe their stability and reliability.

Previously, mnemonics used for diagnosis based on one of these metrics were the use of “SpPin,” which indicated that when specificity is high, a positive prediction confirms the target disease, and “SnNout,” which indicated that when sensitivity is high, a negative result rules it out. However, existing research has shown that this is not entirely reliable [49,50]. Therefore, we recommend that studies that train and test ML and/or DL techniques display multiple metrics.

The dataset on which the algorithms were trained consisted of 28,000 records, of which 80% were used as the training set (22,400) and 20% for the test set (5600). All models underwent 10-fold cross-validation. According to statistical analyses, it was possible to reduce the features from 67 to 20; however, given the context in which the model is being used, it is not advisable to lose medical information without clinical and ethical validation. Even so, the models were trained with both the group of sixty-seven and the group of twenty features. The results reported below correspond to the 67 features, as they obtained better performance using Equations (6)–(14), AUC-ROC, and computational time. Although less mention is made of the group of twenty features. Training and testing were executed thirty times for each algorithm; subsequently, their performance was analyzed with different metrics. Their values were averaged, and their standard deviation was calculated. For all the above, the integrated development environment MATLAB v. R2024a was used on the Windows platform, on a PC with Intel (R) Core i5 and 32 GB of RAM.

The evaluation of ML and DL models in the medical field requires a multi-faceted approach. Relying on a single metric can be misleading, especially when making the differential diagnosis of diseases with overlapping symptoms such as Dengue, Zika, and Chikungunya. A comprehensive evaluation is essential for understanding the models’ performance, advantages, and shortcomings. The rationale for the selected metrics is to balance diagnostic accuracy with clinical safety (i.e., minimizing false negatives) and resource efficiency. We categorized the metrics into three functional groups: basic classification performance, diagnostic robustness, and computational efficiency.

#### 3.5.1. Basic Classification and Reliability Metrics

The primary assessment of model performance begins with accuracy (Equation (6)), which calculates the overall proportion of correct predictions across all classes. However, in clinical contexts, accuracy alone does not reflect the model’s reliability in specific cases. Therefore, we utilize Cohen’s Kappa coefficient, a metric of agreement between categorical samples that adjusts for the possibility of agreement occurring by chance. In this study, we use the weighted kappa ($\kappa_{w}$) (Equation (7)) to observe the consistency of the models as if they were multiple evaluators agreeing on a diagnosis.

(6) $$accuracy=\frac{TP+TN}{TP+TN+FP+FN}$$

(7) $$\kappa_{w}=1-\frac{\sum_{k=1}^{N}\sum_{n=1}^{N}w_{kn}\cdot \;{fo}_{kn}}{\sum_{k=1}^{N}\sum_{n=1}^{N}w_{kn}\cdot {fe}_{kn}}$$

where TP, TN, FP, and FN refer to true positives, true negatives, false positives, and false negatives, respectively. In Equation (7), $\kappa_{w}$ represents the weighted agreement beyond chance, where a value of 1 indicates perfect agreement, and 0 denotes performance no better than random guessing. The term $w_{kn}$ signifies the weighting factor applied to a cell $\left(k,\;n\right)$ to penalize specific disagreements, particularly clinically critical misclassifications, while $fo_{kn}$ and $fe_{kn}$ represent the observed and expected frequencies in the confusion matrix, respectively, under the assumption of independence, [67]. Finally, N defines the number of diagnostic categories Dengue, Zika, Chikungunya, and Influenza, with k and n serving as the indices for the actual and predicted classes, [68]. For uniform kappa $\kappa_{0}$, the value $w_{kn}$ is simplified in Equation (8).

(8) $$w_{kn}=\left\{\begin{array}{cc} 0 & if\;k=n\\ 1 & if\;k\neq n \end{array}\right.$$

For linear kappa, $\kappa_{1},$ the value $w_{kn}$ is calculated using Equation (9).

(9) $$w_{kn}=\frac{\left|k-n\right|}{N-1}$$

For quadratic kappa, $\kappa_{2}$, the value $w_{kn}$ is computed using Equation (10).

(10) $$w_{kn}=\frac{{\left(k-n\right)}^{2}}{{\left(N-1\right)}^{2}}$$

#### 3.5.2. Diagnostic Precision and Sensitivity

In differential diagnosis, there is a critical trade-off between precision and sensitivity. Precision measures the proportion of positive predictions that were truly correct, ensuring that patients are not misdiagnosed with a disease they do not have. Conversely, sensitivity (or recall) measures the model’s ability to identify all positive instances correctly, which is vital in ensuring that no infected patient is overlooked. To balance these inversely related metrics, we employ the F1-score, calculated as their harmonic mean, providing a single measure of the model’s diagnostic effectiveness. Additionally, specificity is used to measure the model’s ability to correctly reject patients who are not infected, a key factor in reducing unnecessary clinical interventions.

(11) $$precision=\frac{TP}{TP+FP}$$

(12) $$sensitivity=\frac{TP}{TP+FN}$$

(13) $$specificity=\frac{TN}{TN+FP}$$

(14) $$F1=2\cdot \frac{precision\cdot sensitivity}{precision+sensitivity}$$

#### 3.5.3. Discrimination Capacity and Efficiency

To evaluate the models across various classification thresholds, we use the ROC (Receiver Operating Characteristic) curve. The AUC-ROC (Area Under the Curve) represents the probability that the model correctly distinguishes a random positive example from a random negative one, offering a threshold-independent measure of discrimination capacity. Finally, Computing Time is monitored as a key performance indicator of efficiency. This is particularly relevant for future implementation in real-time clinical support tools or mobile applications, where rapid diagnostic results are essential for patient prognosis.

## 4. Results

### 4.1. Statistical Analysis

**Figure 2:**
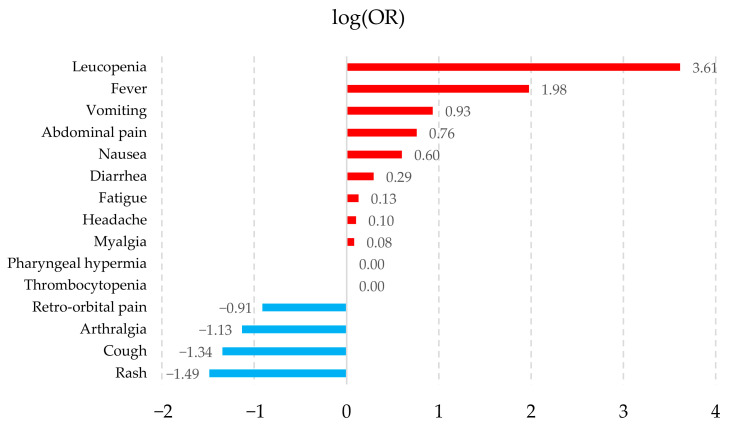
Dengue symptom association graph. The fifteen symptoms are shown with a highly significant *p*-value (<0.05) according to their log(OR). Positive associations are shown in red and negative associations in blue.

**Figure 3:**
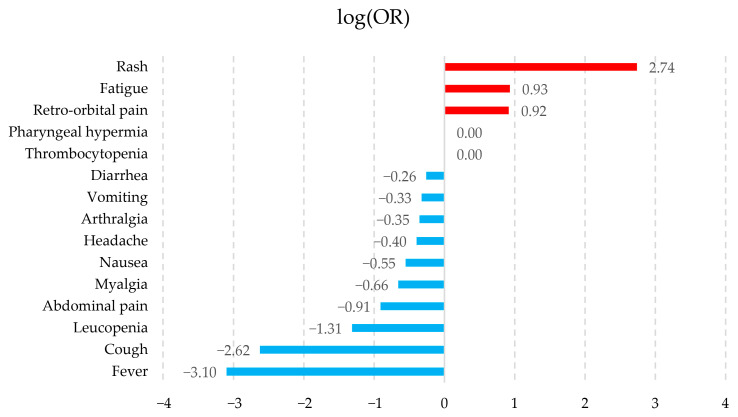
Zika symptom association graph. The fifteen symptoms are shown with a highly significant *p*-value (<0.05) according to their log(OR). Positive associations are shown in red and negative associations in blue.

**Figure 4:**
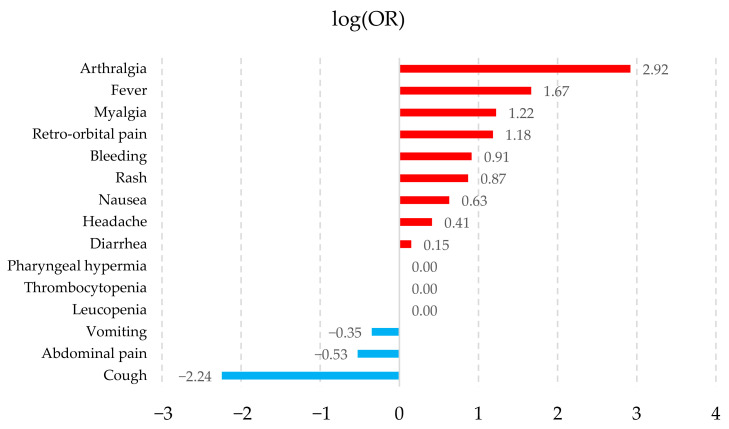
Chikungunya symptom association graph. The fifteen symptoms are shown with a highly significant *p*-value (<0.05) according to their log(OR). Positive associations are shown in red and negative associations in blue.

**Figure 5:**
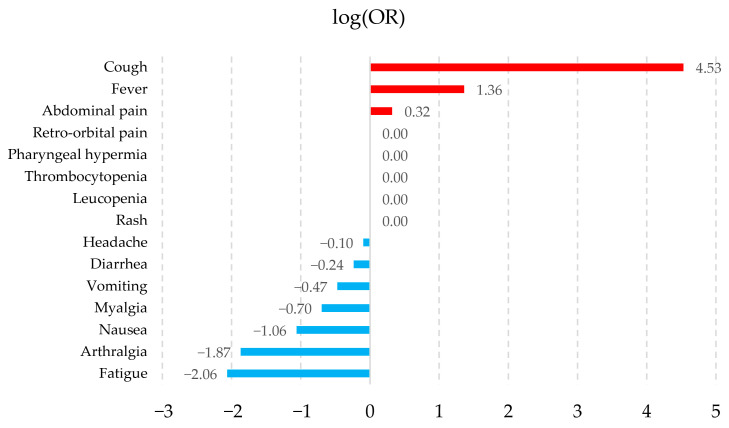
Influenza symptom association graph. The fifteen symptoms are shown with a highly significant *p*-value (<0.05) according to their log(OR). Positive associations are shown in red and negative associations in blue.

The association of symptoms with the pathologies was calculated by logarithmic transformation of their ORs (logOR), which allows for a better visualization of those symptoms that tend to be associated with a particular pathology. Figure 2, Figure 3, Figure 4 and Figure 5 show the symptoms with the positive association in red and those with the negative association in blue. The fifteen symptoms with a highly significant *p*-value (<0.05) are shown for each pathology; however, of the 67 symptoms observed, 65 had a statistically significant result.

The symptoms observed in Figure 2, Figure 3, Figure 4 and Figure 5 show that the observed behavior of symptoms in the created dataset is similar to those already known for each pathology. Furthermore, graphically displaying the symptoms with a statistically significant value allows us to demonstrate that, although these diseases share common signs and symptoms, they also possess some that are more strongly associated with each pathology.

**Figure 6:**
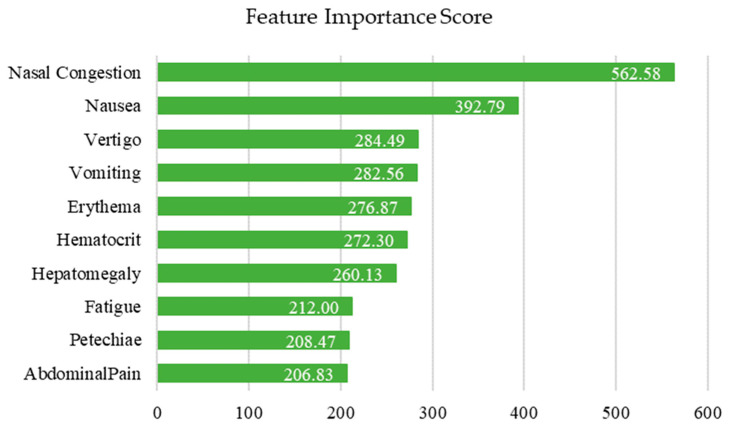
Feature Importance Scores. Symptoms with a score above 200, calculated by the Chi-square algorithm, are shown.

Similarly, to determine the potential influence of each symptom on the models, a Feature Importance test was performed using the Chi-square algorithm. In the test, a very low or near-zero value may indicate that the feature could be eliminated to simplify the model. Scores ranging from 0.9 to just over 500 were observed, and 18 symptoms were classified as infinite. Ultimately, the decision was made to use all symptom data, as out of the total of 67 symptoms, only six showed very low values (score less than 10). Figure 6 shows those symptoms with a score above 200 (infinite symptoms are not shown).

Features with an infinity value were not shown in the figure, as this type of value could be obtained due to the high prevalence of these features in the dataset. The 10 features with the highest scores were shown. These ten features, according to the Feature Importance test, could be the triggers for discriminating between each class in the records.

**Figure 7:**
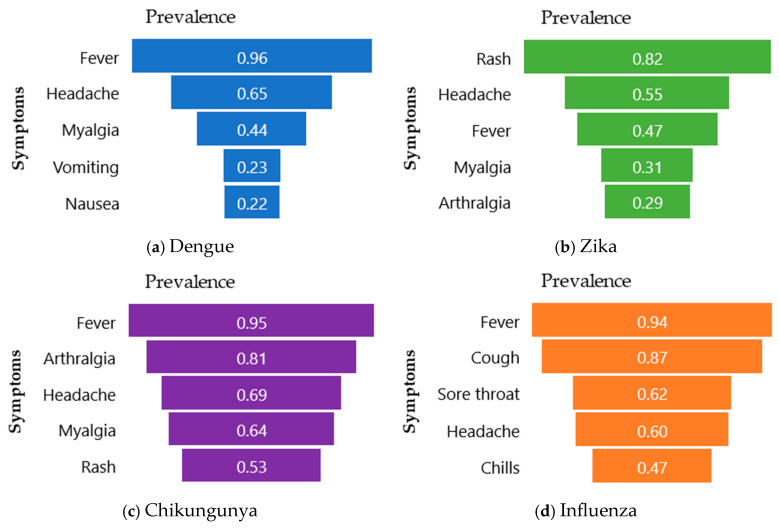
Symptom frequency. (**a**) Most prevalent symptoms in Dengue, (**b**) most prevalent symptoms in Zika, (**c**) most prevalent symptoms in Chikungunya, and (**d**) most prevalent symptoms in Influenza.

Lastly, the frequency of each symptom was quantified from this dataset. Figure 7 shows the five most frequent symptoms for each pathology.

Observing the prevalence of symptoms is the simplest way to demonstrate symptom behavior. The results obtained can be compared with real-world datasets.

The foregoing statistical analyses—log(OR), Feature Importance, and Prevalence analyses—aimed to understand the behavior of the created synthetic dataset. Results show the dataset aligns with PAHO guidelines [25], with symptoms like fever, nausea, rash, headache, myalgia, petechiae, and leukopenia. Similar findings come from Ecuadorian data [69], indicating a high prevalence of symptoms such as myalgia, arthralgia, lethargy, headache, nausea, abdominal pain, and retro-orbital pain, aligning with real-world data. In the same way, the synthetic dataset’s validity is confirmed, since it is similar to the one reported by Batista et al. [70], studying 763 patients from Rio de Janeiro, who found fever, headache, arthralgia, and myalgia common in Dengue, Zika, and Chikungunya.

### 4.2. Model Performance

**Figure 8:**
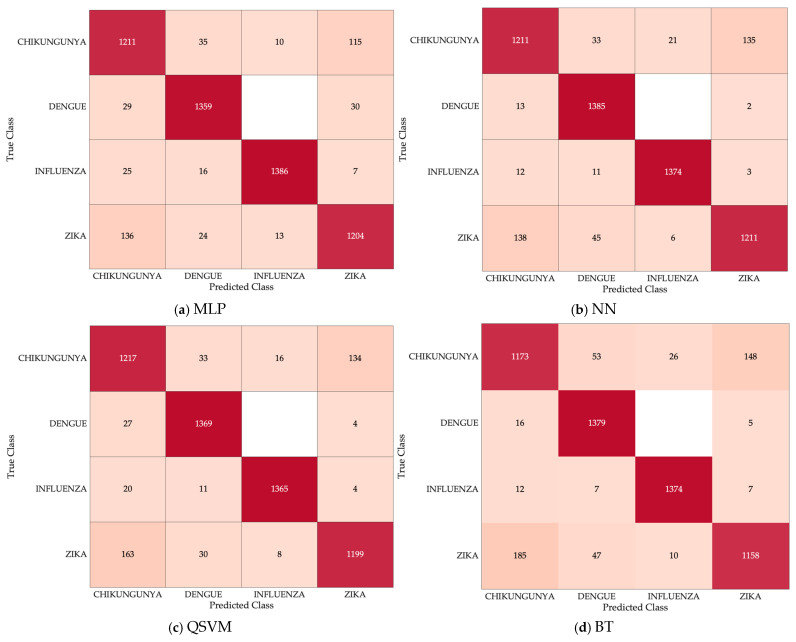
Multiclass confusion matrices. A confusion matrix resulting from each model is shown, the prediction that are correctly classified are shown in dark red. MLP, Multi-Layer Perceptron; NN, Narrow Neural Network; QSVM, Quadratic Support Vector Machine; BT, Bagged Tree.

For each class predicted by the model, there is a true class. Confusion matrices are used to visualize this; the performance of the models can be evaluated using this table. Each row of the confusion matrix presents the instances of the true classes, and each column, the instances of the predicted classes. Therefore, the diagonal of this matrix represents the predictions correctly classified by the model. With the data from these representations, various calculations can be performed to evaluate performance. In the case of multiclass classification, this configuration is extended. Figure 8 shows a multiclass confusion matrix extracted from each model, a result within 30 iterations of each.

The numbers observed on the diagonals of the confusion matrices indicate that the models exhibited very similar behavior among themselves, as well as among the classes. This is probably because the type of relationships observed between the features by the models is general enough that, regardless of the type of mathematical algorithm of the model, the results are similar.

Each of the thirty iterations of each model was evaluated using the following performance metrics: accuracy, precision, sensitivity, specificity, F1-score, AUC-ROC, and Cohen’s kappa. Below, the averaged results for each model are presented.

It is important to mention that prior to these models, other algorithms tested were Linear Discriminant Analysis (LDA), Medium K-Nearest Neighbors (MKNN), and Random Forest (RF), which obtained the following accuracy values of 0.88, 0.90, and 0.87, respectively. Therefore, this first metric allowed us to select the models with the highest values. Table 3 shows the results of different algorithms in a single iteration.

**Figure 9:**
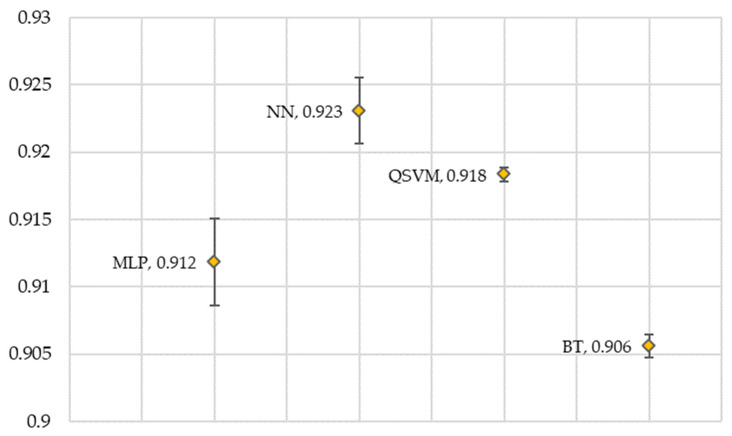
Accuracy of the models. All models were run 30 times each; the graph shows the average accuracy of each model. MLP, Multi-Layer Perceptron; NN, Narrow Neural Network; QSVM, Quadratic Support Vector Machine; BT, Bagged Tree.

On balanced datasets, accuracy serves as a coarse-grained quality measure. The four models tested obtained average results above 0.90 (Figure 9). Furthermore, the very low standard deviation observed in the results indicates low dispersion. As a result, we can see very stable behavior across all models.

An accuracy greater than 0.90 indicates that the models correctly classify more than 90% of the observations; however, in a multiclass scenario, this metric can be misleading. Consequently, it is best to supplement it with additional metrics for each class. This way, it can be determined whether accuracy translates into actual predictive capacity.

**Figure 10:**
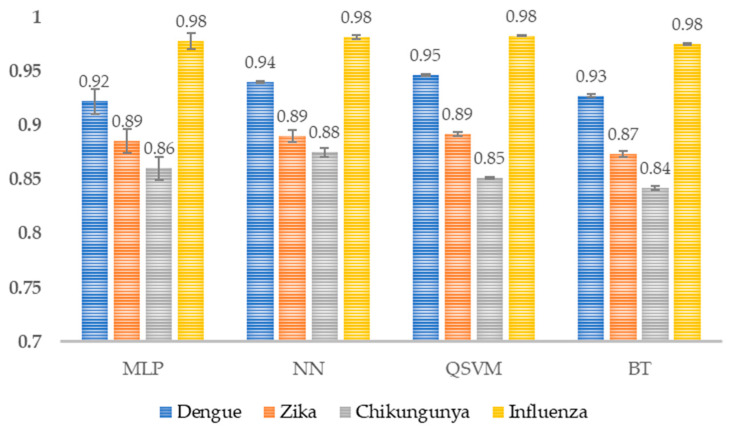
Model precision per class. The average precision per class for each model is shown (*n* = 30). MLP, Multi-Layer Perceptron; NN, Narrow Neural Network; QSVM, Quadratic Support Vector Machine; BT, Bagged Tree.

Scores ranging from 0.84 to 0.98 were obtained for precision, where Chikungunya scored lowest, and Influenza scored highest in all models (Figure 10).

For each class, more than 84% of the instances predicted as positive are correct. This implies a low false positive rate and suggests that the model is reliable when assigning a specific class.

**Figure 11:**
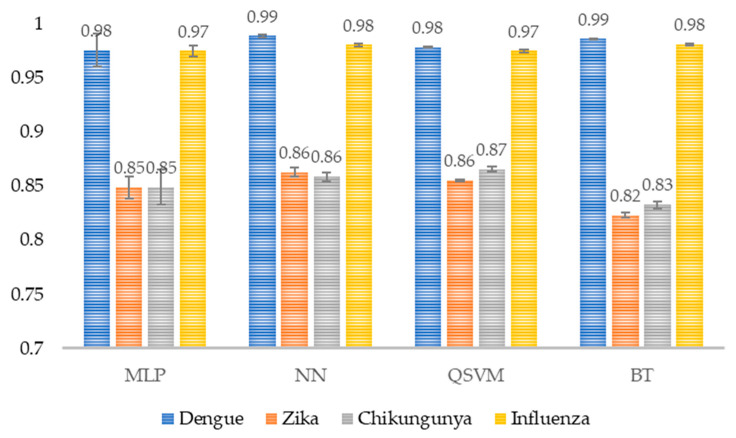
Model sensitivity per class. The average sensitivity per class for each model is shown (*n* = 30). MLP, Multi-Layer Perceptron; NN, Narrow Neural Network; QSVM, Quadratic Support Vector Machine; BT, Bagged Tree.

For sensitivity, the scores obtained exceed 0.82 in each class (Figure 11). Similarly, to precision results, the highest values are observed for Dengue and Influenza, and lower for Zika and Chikungunya. Likewise, its standard deviation is very low.

The model correctly identifies at least 82% of the actual instances in each class. This indicates a good ability to correctly detect positive cases, with a relatively low proportion of false negatives.

**Figure 12:**
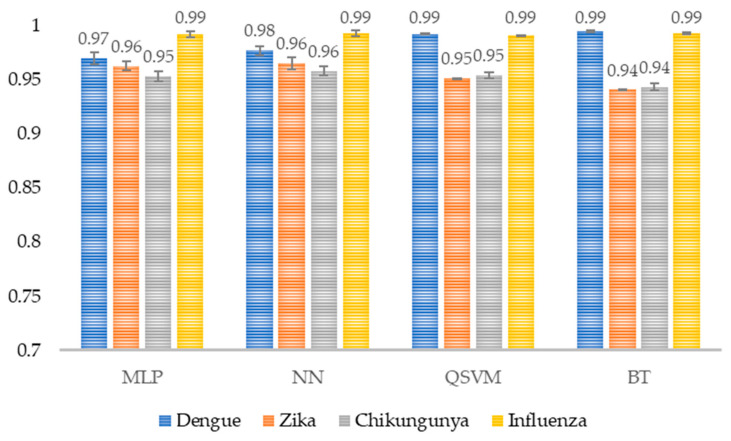
Model specificity per class. The average specificity per class for each model is shown (*n* = 30). MLP, Multi-Layer Perceptron; NN, Narrow Neural Network; QSVM, Quadratic Support Vector Machine; BT, Bagged Tree.

The results yielded values above 0.94 (Figure 12) in the specificity metric, suggesting that these models can recognize a patient who does not have the target disease.

The high specificity reflects that the model distinguishes negative cases very well for each class, avoiding misclassifications into inappropriate classes. This value is particularly relevant in problems where false positives are costly.

**Figure 13:**
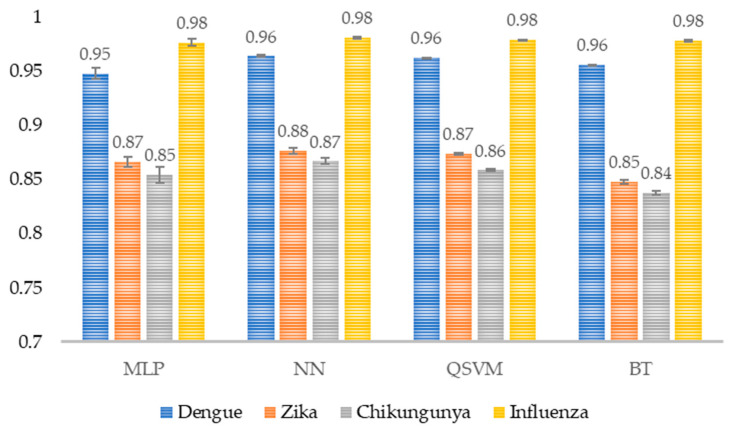
Model F1-score per class. The average F1-score per class for each model is shown (*n* = 30). MLP, Multi-Layer Perceptron; NN, Narrow Neural Network; QSVM, Quadratic Support Vector Machine; BT, Bagged Tree.

In the F1-score, the results obtained exceed 0.84 (Figure 13), indicating that the models are effective at identifying true positives and minimizing the number of false positives and false negatives.

The F1-score combines accuracy and sensitivity, so values above 0.84 indicate that the model maintains a good balance between the two. This suggests that one metric is not being optimized at the expense of the other, but rather that it is being optimized in a balanced and stable manner in the face of potential imbalances between classes.

**Figure 14:**
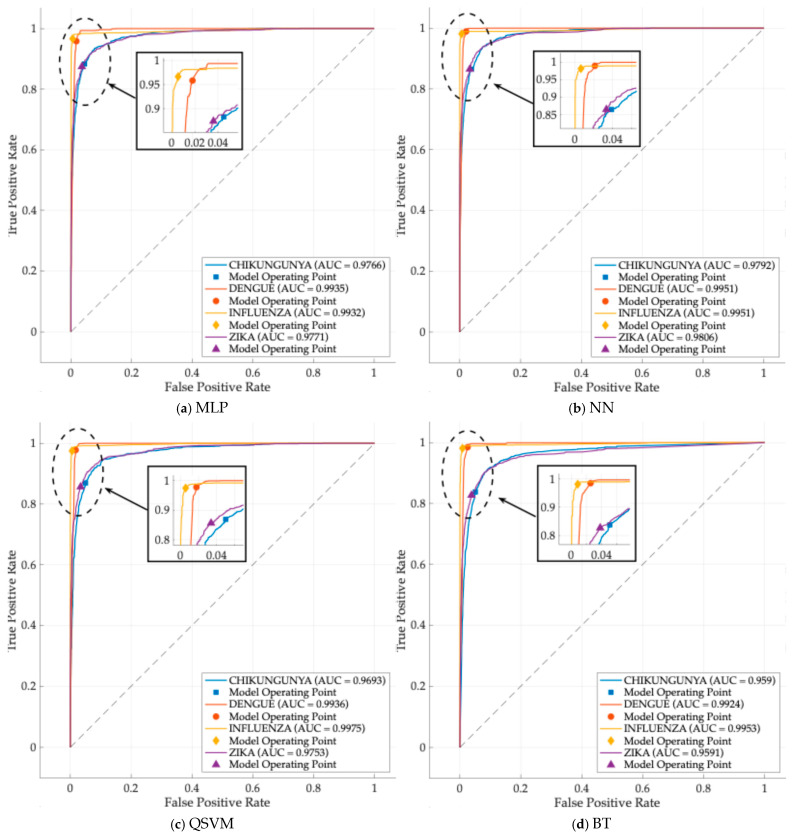
ROC tests for each model. A ROC plot resulting from each model is shown. MLP, Multi-Layer Perceptron; NN, Narrow Neural Network; QSVM, Quadratic Support Vector Machine; BT, Bagged Tree.

**Figure 15:**
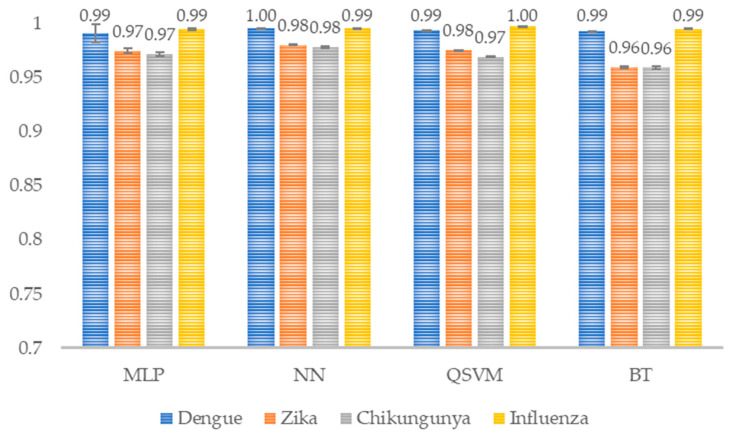
Model AUC per class. The average AUC per class for each model is shown (*n* = 30). MLP, Multi-Layer Perceptron; NN, Narrow Neural Network; QSVM, Quadratic Support Vector Machine; BT, Bagged Tree.

Next, a ROC plot of each model resulting from one of the thirty iterations is shown (Figure 14). The models showed an average AUC above 0.95 (Figure 15) in all classes, indicating good performance.

An AUC greater than 0.96 indicates excellent discrimination between each class and the others. This implies that the model separates the probability distributions between classes very well, even before setting a decision threshold, suggesting that the representation of the predictor variables is highly informative.

**Figure 16:**
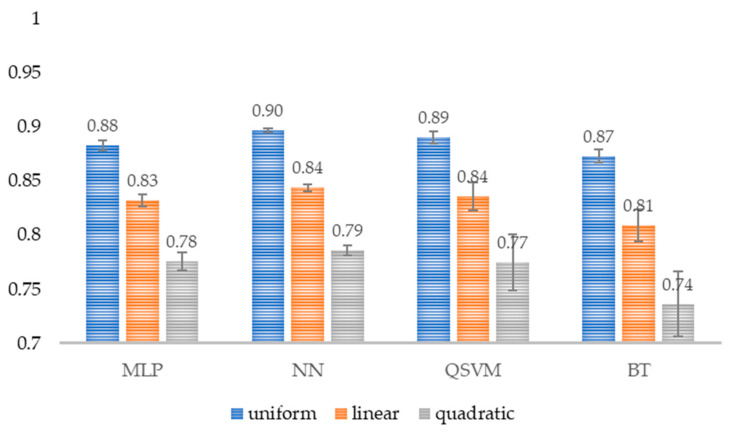
Cohen’s kappa of the models. The different configurations of the average kappa for each model are shown (*n* = 30). MLP, Multi-Layer Perceptron; NN, Narrow Neural Network; QSVM, Quadratic Support Vector Machine; BT, Bagged Tree.

Three results are presented: uniform kappa, linear kappa, and quadratic kappa (Figure 16). The difference between the three weight configurations is the penalty for observed disagreement. However, in any of the configurations, the interpretation remains the same; 1 represents perfect agreement, and 0 represents poor agreement. The kappa values obtained allow us to state that the models exhibit good reliability.

The metric results were supported by a uniform Cohen’s kappa coefficient greater than 0.81, demonstrating almost perfect agreement between the model’s predictions and the actual labels, as Cohen’s kappa corrects for the effect of chance and strengthens the results obtained.

Finally, Table 4 presents all the performance metrics for all the models trained in this work; this represents the arithmetic average of thirty iterations for each model. The training time in seconds was also added. That is, the amount of time the models require to execute based on the amount of data supplied and the arithmetic complexity of each algorithm. Similarly, the metrics for the NN model (Table 5) are presented with the group of 20 features; this model is presented to observe that there is a better performance among the models with 67 features.

In the case of MLP, three groups were created with different numbers of epochs: a first group of 10 iterations with 100 epochs, a second group of 10 iterations with 500 epochs, and a third group of 10 iterations with 1000 epochs.

## 5. Discussion

The results obtained indicate that the synthetic dataset exhibits behavior consistent with the symptomatology described by the PAHO guidelines [26]. For example, in Dengue without warning signs, PAHO reports the following symptoms: fever, nausea or vomiting, rash, headache, retro-orbital pain, myalgia or arthralgia, petechiae, and leukopenia. These symptoms appear in the statistical analyses conducted and similarly demonstrate a strong association with the condition.

Another dataset, collected from sentinel medical centers in Ecuador [71] between November 2013 and September 2017, consists of patients with suspected arboviral infections (Dengue, Zika, and Chikungunya). Their reported findings corroborate a high prevalence of symptoms such as myalgia and arthralgia, lethargy, headache, nausea or vomiting, abdominal pain, and retro-orbital pain—alongside fever—which suggests that our synthetic dataset is analogous to a real-world dataset.

Similarly, Batista et al. [70], in their study of 763 patients confirmed with Dengue, Zika, and Chikungunya (Dengue = 51, Zika = 88, Chikungunya = 624), conducted a distributional analysis of clinical signs and symptoms. The patients were recruited between January 2016 and September 2019 across 23 public health centers in Rio de Janeiro, Brazil. According to their findings, the symptoms with the highest prevalence were as follows: for Dengue—fever, headache, and arthralgia; for Zika—fever, headache, myalgia, and arthralgia; and for Chikungunya—fever, arthralgia, myalgia, and headache. These results allow for a direct comparison with the patterns observed in our synthetic dataset.

In the context of Dengue, Zika, and Chikungunya, Arrubla-Hoyos et al. [38] also implemented ML algorithms to predict these diseases. The research team used the PAHO 2022 guidelines in their models to assign weights to their features and compared their results by evaluating datasets with and without this change in weights. Better performance was observed in models that had weights assigned according to the guidelines. For our part, we allowed the ML and DL model architectures themselves to search for relationships between features, which also showed good performance.

Ozer et al. [39], for their part, evaluated and trained different ML and DL algorithms with notable performance. Their study used these algorithms to analyze two datasets, one that included laboratory tests and the other that did not. Their purpose was to predict when to hospitalize a patient with any of these three diseases. This would be very useful in a triage area, as it would serve as a medical tool for a process that requires speed. The approach of this research team, the use in triage, could be part of a future project for our work; that is, rapid resolution at a critical stage of patient care.

In the work of Abdallah et al. [40], ML algorithms were used to analyze social and environmental features and thus predict the outbreak of these vector-borne diseases. Their models were evaluated using accuracy, precision, sensitivity, F1-score, and AUC metrics. Using this same approach, Georgiades et al. [41] sought to predict future habitats of the species *Aedes* albopictus, a vector of the pathologies described here, using ML models. Their findings predict a global increase in the habitat of this species, suggesting that climate change plays a significant role. This encourages us to consider further analysis of the performance metrics shown here. Analyses such as Local Interpretable Model-agnostic Explanations (LIME) or Shapley Additive Explanations (SHAP) can be implemented to determine the final contribution of each feature to the model results.

The analysis of Dong et al. [42] also used ML algorithms to predict risk factors for outbreaks of these diseases. They analyzed local sociodemographic and climatic features of 2469 municipalities in Mexico. Their analyses show that a better understanding of these microenvironments could help us develop local health and prevention programs. Table 6 compares the aforementioned studies, showing the performance metrics of their ML models versus our results.

Our models, based on their scores, perform better at distinguishing Dengue from other diseases, especially Influenza, used here as a negative control. The numbers show a slight decrease in discrimination between Zika and Chikungunya; nevertheless, we consider this a good performance. Most likely, the models’ reduced ability to distinguish between Zika and Chikungunya is because the cases in the dataset exhibit very similar symptom patterns between these two diseases, suggesting that only one or two features may truly differentiate them. This observation opens a path for future analysis of the relationship between the two diseases.

It is important to mention that a false negative case in Dengue can be risky, as unsuitable treatment can worsen the disease. In the case of Zika, if it is detected during pregnancy, it can help with birth control, as a correlation has been observed between Zika and microcephaly. Given these observations and considering the cited literature, while our results are not as high as others, they are more stable across the four pathologies analyzed. Furthermore, the inclusion of a disease as a negative control is due to the fact that this model could only discriminate between arboviral diseases. However, the complexity of real-life situations means that a patient may present with similar symptoms without suffering from any of these three pathologies, so this is an attempt at approximation.

Similarly, Cohen’s kappa coefficient was added to the metrics, with different configurations, to observe the coefficient of concordance. Scores greater than 0.75 indicate the stability of the models, which lends greater reliability to the performance metrics. Likewise, showing training times allows us to compare the efficiency and performance of the models. This measures computational costs, such as the time and memory required by the models; it should be noted that these can be subject to change, as they depend on factors such as the algorithm, the size of the input data (which in this work was reduced because of coding), the hardware (i.e., processor power and available memory), as well as the language and its compiler. All the above have led to the fact that, for simplicity, only the time taken by the models is displayed.

We can add that reducing the features in our models reduced not only their final performance but also their computational cost which, in this case, is by 7.04% in the model shown. The above is useful in providing a model proposal for computers with compromised benchmarks, i.e., machines with reduced or outdated processors, RAM, and/or graphics cards; this is, of course, at the cost of final performance.

One contribution of this work can be seen in the creation of a synthetic medical dataset. Data collection, processing, and transformation showed results very close to the analysis of real datasets, so it is proposed that, given the scarcity or limitations in obtaining optimal data, as well as class imbalances, which are common in the observation of this type of disease, the use of synthetic data is feasible. Similarly, the binary transformation performed here facilitates the handling of different types of data, which can simplify the models. This could help avoid excessive consumption of computational and energy resources during large-scale implementation. In fact, simplifying the data also allows ML models to better generalize the relationships within the data.

The findings of this work suggest that the data represented using our methodology, along with class balance in training and the inclusion of various performance metrics, provide guidelines for the development of a technological application and its practical application. The results shown are based on the TRIPOD+AI (Transparent Report of a Multivariable Predictive Model for Individual Prognosis or Diagnosis, Artificial Intelligence) declaration methodology, a checklist with 27 detailed elements that aims to align studies with ML models; this helps in the preparation of AI-driven reports [72].

## 6. Limitations

Limitations of our study are that the findings reported here only refer to the performance demonstrated by in silico models. Furthermore, the methodology used to generate a synthetic binary dataset aims to generalize symptoms for global use, but this may oversimplify and require validation by a clinical expert. Similarly, real patient data could be used to validate the predictive capacity of the trained models. It should be noted that this is an effort to improve public health.

Similar ML studies for arboviral diseases face the problem that they work well in laboratory settings but present some difficulties when applied to real patients [39]. In addition, we could overlook important differences in disease manifestations across population groups. By excluding factors such as age, ethnic groups, and geography from our dataset, we could have overlooked how these factors influence symptom patterns [40].

Influenza and arboviral diseases share many symptoms, such as fever and muscle pain, making it difficult to distinguish clinically. Our models easily separated Dengue from Influenza, but struggled with the differences between Zika and Chikungunya. In practice, clinicians often care for patients during outbreaks when multiple similar diseases are circulating simultaneously. While Influenza serves as a reasonable negative control for testing our models, future versions should include other common febrile illnesses, such as bacterial infections, to better reflect the diagnostic complexity that clinicians face in practice [41].

Despite these constraints, this study makes methodological contributions by demonstrating a systematic symptom-encoding framework, a comprehensive multi-algorithm comparison, and a rigorous statistical feature analysis applicable to future real-world studies. The synthetic data generation protocol and analysis code could be valuable for research teams planning similar projects in data-scarce environments. However, we emphasize that no clinical decisions should be based on these synthetic-data-derived models until prospective validation confirms their safety and effectiveness in actual patient populations.

## 7. Conclusions

In this study, a synthetic dataset was created for the pathologies of Dengue, Zika, and Chikungunya. In addition, Influenza was included as a negative control. This synthetic, binary dataset aims to generalize the pathology data, and transforming it into binary data allowed us to standardize the collected data. Subsequently, its behavior was verified through statistical analysis, with results analogous to a real-world dataset. According to the statistical analyses, symptoms strongly associated with the pathologies were found, similar to those reported by the PAHO and other data set. The synthetic dataset was used to train and evaluate ML techniques. The results obtained demonstrate the performance of machine learning and deep learning techniques in the differential diagnosis of Dengue, Zika, and Chikungunya. These techniques have shown promising results. The use of AI techniques could accelerate the early diagnosis process and, consequently, improve the prognosis. In our study, the best results were obtained by the NN model, a simple neural network.

Considering the nature of this synthetic dataset and the stability shown by the trained models—all four pathologies yielded good results—future research could lead us in two main directions. First, it is important to test these models in real-world clinical settings to see how they can support healthcare professionals in their decision-making. Second, future work will explore a wider range of AI techniques, such as using multimodal data (like genetic markers or environmental factors) or testing popular algorithms like XGBoost and auto-encoders to reduce features. Analyzing these additional data types and advanced models will help us understand whether we can improve the accuracy and applicability of predictions in different regions affected by arboviral diseases. If successful, we plan to move to pilot testing with an online platform or application for real-world use. We emphasize that this study and future work are intended to assist healthcare professionals, not replace them, as their expertise and judgment are always essential.

These models should be rigorously evaluated using various performance metrics. We recommend repeating the experiment several times to observe their behavior. Once implemented, these models can be evaluated with field data to observe their practical implications and true predictive capacity. It is important to remember that these types of diseases can arise from sociodemographic factors; these factors were not considered in this study. Our data collection methodology helped us minimize dataset problems, such as unbalanced datasets.

These results also allow us to explore future lines of research. If the performance is adequate in the in silico experiments, it encourages us to explore its potential by including real-world datasets and observing whether it maintains the same behavior.

## Figures and Tables

**Table 1:** Sixty-seven signs and symptoms observed in the data collected from the diseases of Dengue, Zika, Chikungunya, and Influenza, classified by the physiological system they affect.

Physiological System	Signs and Symptoms
General system	Fever, Chills, Fatigue, Malaise, Lethargy, Anorexia, General pain, Edema
Gastrointestinal system	Nausea, Vomiting, Diarrhea, Constipation, Abdominal pain, Hepatomegaly, Melena, Mouth sore, Odynophagia
Respiratory system	Cough, Dyspnea, Tachypnea, Wheezing, Sputum, Sneezing, Rhinorrhea, Nasal Congestion, Rhinitis, Sore throat, Pharyngeal hyperemia, Adenoids, Otitis
Neurological system	Headache, Impaired consciousness, Seizures, Dizziness, Vertigo, Paresthesia, Sleeping disorder, Concentration disorder, Anosmia, Ageusia, Dysgeusia
Dermatological system	Rash, Maculopapular rash, Erythema, Petechiae, Pruritus, Cutaneous hypersensitivity
Musculoskeletal system	Myalgia, Arthralgia, Low back pain
Cardiovascular system	Tachycardia, Low SBP, Shock, Plasma leakage, Chest pain
Hematological system	Bleeding, Mucosal bleeding, Leucopenia, Thrombocytopenia, Hematocrit, Lymphadenopathy
Renal system	AKI, Oliguria, Dysuria
Ophthalmological system	Retro-orbital pain, Conjunctivitis, Photophobia

**Table 2:** Contingency Counts Formal Derivation.

**Count**	Patient Status	Formal Summation
a	$\mathrm{Both}\;j_{1}$$\;\mathrm{and}\;j_{2}$ are present (1).	a= $\sum_{i=1}^{m}I_{\left\{x_{i},j_{1}=1\;and\;x_{i,j_{2}}=1\right\}}$
b	$j_{1}$$\;\mathrm{is}\;\mathrm{present}\;(1)\;\mathrm{and}\;j_{2}$ are absent (0).	b= $\sum_{i=1}^{m}I_{\left\{x_{i},j_{1}=1\;and\;x_{i,j_{2}}=0\right\}}$
c	$j_{1}$$\;\mathrm{is}\;\mathrm{absent}\;(0)\;\mathrm{and}\;j_{2}$ are present (1).	c= $\sum_{i=1}^{m}I_{\left\{x_{i},j_{1}=0\;and\;x_{i,j_{2}}=1\right\}}$
d	$\mathrm{Both}\;j_{1}$$\;\mathrm{and}\;j_{2}$ are absent (0).	d= $\sum_{i=1}^{m}I_{\left\{x_{i},j_{1}=0\;and\;x_{i,j_{2}}=0\right\}}$

**Table 3:** Accuracy of different algorithms.

Model	Acc (Val)	Acc (Test)	Training Time (s)
MLP	0.92	0.92	157.6
NN	0.92	0.92	308.23
QSVM	0.92	0.92	289.75
BT	0.91	0.91	209.29
LDA	0.89	0.88	46.08
MKNN	0.89	0.90	132.4
RF	0.86	0.87	63.99

This is the first iteration of several algorithms to choose those with the best scores. Acc, Accuracy; Val, Validation; sec, Seconds; MLP, Multi-Layer Perceptron; NN, Narrow Neural Network; QSVM, Quadratic Support Vector Machine; BT, Bagged Tree; LDA, Linear Discriminant Analysis; MKNN, Medium K-Nearest Neighbors; RF, Random Forest.

**Table 4:** Performance metrics of the trained models.

Model	Acc	Class	Precc	Sen	Spec	F1	AUC	$\kappa_{w}$	Time (s)
NN	**0.923**	DEN	0.94	0.99	0.98	**0.96**	**1.00**	$\kappa_{0}$ = **0.90**	284
		ZIK	0.89	**0.86**	**0.96**	**0.88**	**0.98**	$\kappa_{1}$ = **0.84**	
		CHI	**0.88**	0.86	**0.96**	**0.87**	**0.98**	$\kappa_{2}$ = **0.79**	
		FLU	0.98	**0.98**	0.99	**0.98**	0.99		
QSVM	0.918	DEN	**0.95**	0.98	0.99	0.96	0.99	$\kappa_{0}$ = 0.89	331
		ZIK	**0.89**	0.86	0.95	0.87	0.98	$\kappa_{1}$ = 0.84	
		CHI	0.85	**0.87**	0.95	0.86	0.97	$\kappa_{2}$ = 0.77	
		FLU	**0.98**	0.97	0.99	0.98	**1.00**		
BT	0.906	DEN	0.93	0.99	**0.99**	0.96	0.99	$\kappa_{0}$ = 0.87	**235**
		ZIK	0.87	0.82	0.94	0.85	0.96	$\kappa_{1}$ = 0.81	
		CHI	0.84	0.83	0.94	0.84	0.96	$\kappa_{2}$ = 0.74	
		FLU	0.98	0.98	0.99	0.98	0.99		
MLP 100 Epochs	0.913	DEN	0.92	0.98	0.97	0.95	0.99	$\kappa_{0}$ = 0.88	520
		ZIK	0.88	0.85	0.96	0.86	0.97	$\kappa_{1}$ = 0.83	
		CHI	0.86	0.85	0.95	0.86	0.97	$\kappa_{2}$ = 0.77	
		FLU	0.98	0.98	0.99	0.98	0.99		
MLP 500 Epochs	0.911	DEN	0.92	0.97	0.97	0.95	0.99	$\kappa_{0}$ = 0.88	2611
		ZIK	0.88	0.85	0.96	0.87	0.97	$\kappa_{1}$ = 0.83	
		CHI	0.86	0.85	0.95	0.85	0.97	$\kappa_{2}$ = 0.77	
		FLU	0.98	0.97	0.99	0.97	0.99		
MLP 1000 Epochs	0.910	DEN	0.91	**0.99**	0.96	0.95	0.99	$\kappa_{0}$ = 0.88	5126
		ZIK	0.89	0.85	0.96	0.87	0.98	$\kappa_{1}$ = 0.83	
		CHI	0.86	0.83	0.95	0.85	0.97	$\kappa_{2}$ = 0.77	
		FLU	0.98	0.97	**0.99**	0.98	0.99		

The data displayed corresponds to the arithmetic average of thirty iterations for each model. The highest values in each metric have been highlighted in bold. MLP, Multi-Layer Perceptron; NN, Narrow Neural Network; QSVM, Quadratic Support Vector Machine; BT, Bagged Tree; DEN, Dengue; ZIK, Zika; CHI, Chikungunya; FLU, Influenza; Acc, Accuracy; Precc, Precision; Sen, Sensitivity; Spec, Specificity; F1, F1-Score; AUC, Area Under the Curve; $\kappa_{w}$, Cohen’s Kappa; $\kappa_{0}$, Uniform Kappa; $\kappa_{1}$, Linear Kappa; $\kappa_{2}$, Quadratic Kappa.

**Table 5:** Performance metrics of the NN model in the group of twenty features.

Model	Acc	Class	Precc	Sen	Spec	F1	AUC	$\kappa_{w}$	Time (s)
NN	0.897	DEN	0.93	0.95	0.98	0.94	0.99	$\kappa_{0}$ = 0.86	264
		ZIK	0.87	0.82	0.96	0.85	0.96	$\kappa_{1}$ = 0.80	
		CHI	0.8	0.84	0.93	0.83	0.96	$\kappa_{2}$ = 0.74	
		FLU	0.98	0.97	0.99	0.98	0.99		

The data displayed corresponds to the arithmetic average of ten iterations for the NN model. NN, Narrow Neural Network; DEN, Dengue; ZIK, Zika; CHI, Chikungunya; FLU, Influenza; Acc, Accuracy; Precc, Precision; Sen, Sensitivity; Spec, Specificity; F1, F1-Score; AUC, Area Under the Curve; $\kappa_{w}$, Cohen’s Kappa; $\kappa_{0}$, Uniform Kappa; $\kappa_{1}$, Linear Kappa; $\kappa_{2}$, Quadratic Kappa.

**Table 6:** Comparison of studies with a focus on AI. It shows how Machine Learning techniques can be used for different purposes. The table compares the metrics of the best model in each study.

Study	Purpose	Acc	Precc	Sen	Spec	F1	AUC	$\kappa_{w}$	AI Model
Arrubla-Hoyos et al. [38]	Predict Disease	0.99	DEN 0.98 ZIK 0.99 CHI 0.99	DEN 0.99 ZIK 0.98 CHI 1.00	0.99	DEN 0.990 ZIK 0.989 CHI 0.999	--	--	RF
Ozer et al. [39]	Predict Hospitalization	1.00	--	1.00	1.00	--	1.00	--	LSTM-TL
Abdallah et al. [40]	Predict Outbreaks	DEN -- ZIK 0.96 CHI 0.93	DEN -- ZIK 0.70 CHI 0.57	DEN -- ZIK 0.86 CHI 0.63	--	DEN -- ZIK 0.78 CHI 0.63	DEN -- ZIK 0.91 CHI 0.79	--	EM
Georgiades et al. [41]	Predict Vector habitat	--	--	0.86	--	0.91	--	--	XGB
Dong et al. [42]	Predict Local Outbreaks	DEN 0.93 ZIK 0.99 CHI 0.99	DEN 0.86 ZIK 0.76 CHI 0.87	DEN 0.78 ZIK 0.61 CHI 0.59	--	DEN 0.81 ZIK 0.65 CHI 0.65	--	--	XGB
Our study	Predict Disease	0.92	DEN 0.94 ZIK 0.89 CHI 0.88 FLU 0.98	DEN 0.99 ZIK 0.86 CHI 0.86 FLU 0.98	DEN 0.98 ZIK 0.96 CHI 0.96 FLU 0.99	DEN 0.96 ZIK 0.88 CHI 0.87 FLU 0.98	DEN 1.00 ZIK 0.98 CHI 0.98 FLU 0.99	$\kappa_{0}$ 0.90 $\;\kappa_{1}$ 0.84 $\;\kappa_{2}$ 0.79	NN

Acc, Accuracy; Precc, Precision; Sen, Sensitivity; Spec, Specificity; F1, F1-Score; AUC, Area Under the Curve; $\kappa_{w}$, Cohen’s kappa; DEN, Dengue; ZIK, Zika; CHI, Chikungunya; FLU, Influenza; $\kappa_{0}$, Uniform Kappa; $\kappa_{1}$, Linear Kappa; $\kappa_{2}$, Quadratic Kappa; RF, Random Forest; LSTM-TL, Long Short-Term Memory Network with Transfer Learning; EM, Ensemble model; XGB, XGBoost; NN, Narrow Neural Network.

## Data Availability

The raw data supporting the conclusions of this article will be made available by the authors on request.

## Abbreviations

The following abbreviations are used in this manuscript:

ML	Machine learning
PAHO	Pan American Health Organization
DL	Deep learning
LSTM	Long short-term memory
AHP	Analytic hierarchy process
MALDI-TOF	Matrix-assisted laser desorption ionization Time-of-flight
AI	Artificial intelligence
DENV	Dengue virus
WHO	World Health Organization
NSAID	Nonsteroidal anti-inflammatory drug
ZIKV	Zika virus
CHIV	Chikungunya virus
FDA	Food and Drug Administration
ROC	Receiver operating characteristic
AUC	Area under the curve
MLP	Multi-layer perceptron
NN	Narrow neural network
QSVM	Quadratic support vector machine
BT	Bagged tree
EM	Ensemble model
XGB	XGBoost

## Appendix A

A total of 125 studies were collected for the synthetic dataset: 50 studies for Dengue [72,73,74,75,76,77,78,79,80,81,82,83,84,85,86,87,88,89,90,91,92,93,94,95,96,97,98,99,100,101,102,103,104,105,106,107,108,109,110,111,112,113,114,115,116,117,118,119,120,121,122,123], 33 for Zika [15,124,125,126,127,128,129,130,131,132,133,134,135,136,137,138,139,140,141,142,143,144,145,146,147,148,149,150,151,152,153,154], 31 for Chikungunya [149,155,156,157,158,159,160,161,162,163,164,165,166,167,168,169,170,171,172,173,174,175,176,177,178,179,180,181,182,183,184], and 11 for Influenza [185,186,187,188,189,190,191,192,193,194,195]. Table A1 lists representative symptoms, the number of symptoms per category, original data source types, geographical origins of source studies, and corresponding citations from the literature review.

**Table A1:** Synthesis of clinical features by physiological system.

Clinical Category	Representative Symptoms and Signs (Data Vectors)	No. of Symptoms	Data Source Type/Repository	Entities and Geographical Origin	Studies
Systemic & Neurological	“Fever, Headache, Myalgia, Arthralgia, Retro-orbital pain, Chills, Fatigue, Photophobia, Impaired consciousness, Seizures, Dizziness, Vertigo, Malaise.”	14	Primary Clinical Datasets & Hospital Records (No public repository specified for most; data available upon request)	“Karachi Children’s Hosp. (PK), Hosp. de Niños (AR), Hosp. Gral. Culiacán (MX), Nigeria, Saudi Arabia, Bangladesh, India, Nepal, Brazil, Guatemala, Puerto Rico, USA, Panama, Cambodia, Maldives, Nicaragua, Peru, Mexico, Colombia, Singapore, Korea, Reunion Island, Thailand.”	[72,73,74,75,76,77,78,79,80,81,82,83,84,85,86,87,88,89,90,91,92,93,94,95,96,97,124,125,126,127,128,129,130,131,132,133,134,135,136,137,138,139,140,141,142,143,144,145,146,147,148,149,150,151,152,153,154,155,156,158,159,160,161,162,163,164,165,166,167,168,169,170,171,172,173,174,175,176,177,178,179,180,181,182,183,184,185,186,187]
Gastrointestinal	“Abdominal pain, Nausea, Vomiting, Diarrhea, Hepatomegaly, Anorexia, Edema, Melena, Mouth sore, Odynophagia.”	11	Public Health Surveillance Datasets (CDC/PAHO/MoH reports; no structured repository)	“CDC Laredo, Ministry of Health Reports (Colombia, Bangladesh), Northern India Centers, Brazil, Guatemala, Puerto Rico, Nicaragua, Peru, Mexico, Cambodia, Thailand.”	[98,99,100,101,102,103,104,105,106,107,157,165,180,183,185]
Dermatological & Musculoskeletal	“Maculopapular rash, Pruritus, Edema, Conjunctivitis (non-purulent), Rash, Erythema, Cutaneous hypersensitivity, Arthralgia, Myalgia, Low back pain.”	12	Open-Access Health Repositories (MMWR/CDC; VA Healthcare datasets available upon request)	“MMWR (CDC), National Surveillance (Brazil, Guatemala), VA Healthcare (USA), Peru, Nicaragua, Maldives, Bangladesh, India, Colombia, Singapore, Korea, Reunion Island.”	[108,109,110,111,112,113,114,115,147,157,158,159,170,171,172,175,180,181,182]
Hemorrhagic & Hematological	“Petechiae, Bleeding, Thrombocytopenia, Leukopenia, High hematocrit, Mucosal bleeding, Plasma leakage, Lymphadenopathy, AKI, Dysuria.”	13	Critical Care & ICU Case Series (No public repository; clinical records internal)	“Tertiary Care Units in Nepal, Thailand, Vietnam, Saudi Arabia, India, Brazil, Guatemala, Puerto Rico, Colombia, Bangladesh.”	[116,117,118,119,120,121,122,164,173,176,179]
Respiratory & Others	“Cough, Sore throat, Rhinorrhea, Nasal congestion, Dyspnea, Chest pain, Wheezing, Lymphadenopathy, Oliguria, Sneezing, Sputum, Tachypnea, Pharyngeal hyperemia, Adenoids, Otitis, Anosmia, Ageusia, Dysgeusia.”	17	Sentinel Surveillance Datasets (Some available via CDC FluView; others upon request)	“Global H1N1 Datasets (Guatemala, India, Taiwan), Puerto Rico Sentinel Sites, Cambodia, Pakistan, USA, UK, Thailand, Bangladesh, Mexico.”	[123,124,125,126,127,128,129,130,131,188,189,190,191,192,193,194,195]
